# Characterization of C_30_ carotenoid and identification of its biosynthetic gene cluster in *Methylobacterium extorquens* AM1

**DOI:** 10.1016/j.synbio.2023.08.002

**Published:** 2023-08-09

**Authors:** Xu-Hua Mo, Yu-Man Sun, Yu-Xing Bi, Yan Zhao, Gui-Hong Yu, Ling-ling Tan, Song Yang

**Affiliations:** a School of Life Sciences, Qingdao Agricultural University, 700 Changcheng Road, Qingdao, Shandong, 266109, China; b Shandong Province Key Laboratory of Applied Mycology, Qingdao Agricultural University, 700 Changcheng Road, Qingdao, Shandong, 266109, China; c Qingdao International Center on Microbes Utilizing Biogas, Qingdao Agricultural University, Qingdao, Shandong Province, China

**Keywords:** C_30_ carotenoid, *Methylobacterium extorquens*, Biosynthetic gene cluster, Glycosyltransferase, Acyltransferase

## Abstract

*Methylobacterium* species, the representative bacteria distributed in phyllosphere region of plants, often synthesize carotenoids to resist harmful UV radiations. *Methylobacterium extorquens* is known to produce a carotenoid pigment and recent research revealed that this carotenoid has a C_30_ backbone. However, its exact structure remains unknown. In the present study, the carotenoid produced by *M. extorquens* AM1 was isolated and its structure was determined as 4-[2-*O*-11*Z*-octadecenoyl-β-glucopyranosyl]-4,4′-diapolycopenedioc acid (**1**), a glycosylated C_30_ carotenoid. Furthermore, the genes related to the C_30_ carotenoid synthesis were investigated. Squalene, the precursor of the C_30_ carotenoid, is synthesized by the co-occurrence of META1p1815, META1p1816 and META1p1817. Further overexpression of the genes related to squalene synthesis improved the titer of carotenoid **1**. By using gene deletion and gene complementation experiments, the glycosyltransferase META1p3663 and acyltransferase META1p3664 were firstly confirmed to catalyze the tailoring steps from 4,4′-diapolycopene-4,4′-dioic acid to carotenoid **1**. In conclusion, the structure and biosynthetic genes of carotenoid **1** produced by *M. extorquens* AM1 were firstly characterized in this work, which shed lights on engineering *M. extorquens* AM1 for producing carotenoid **1** in high yield.

## 1 Introduction

The lipophilic natural carotenoids belong to a class of isoprenoid derivatives. So far, more than 1100 carotenoids have been isolated from various plants and microorganisms [1]. The carotenoids have multiple conjugated double bonds, which enable two essential features of carotenoids: the light-harvesting capability and powerful anti-oxidant effect by quenching of free radicals, singlet oxygen and reactive oxygen species [1]. Carotenoids are widely used as colorants and additives in food industry [2]. Additionally, for human beings, carotenoids are shown to inhibit cancer cells, serve as antioxidants, and enhance the immune response to decrease the risk of multiple diseases, especially eye diseases [[3], [4], [5]]. Structurally, carotenes such as lycopene, β-carotene, and α-carotene are hydrocarbons that can be linear or cyclized, while xanthophylls like lutein and astaxanthin are oxygenated derivatives of carotenes with hydroxyl, keto, or epoxy groups [[6], [7], [8]]. Most of carotenoids are typical C_40_ based derivatives, which are produced by photosynthetic bacteria and plants. Whereas for several non-photosynthetic bacteria species such as *Rubritalea squalenifaciens* [9], *Staphylococcus aureus* [10], *Bacillus firmus* [11], *Streptococcus faecium* [12], *Methylomonas* sp. strain 16a [13], *Methylobacterium* spp. strains [14], and *Planococcus* spp. strains [[15], [16], [17], [18]], the unique C_30_ based carotenoids were identified.

The biosynthetic pathway of C_30_ carotenoid staphyloxanthin, a virulence factor generated by the conditional pathogen *S. aureus*, was identified by employing a combination of gene deletion and heterologous expression experiments, which includes a cluster of five genes *crtMNPQO* and an individual gene *aldH* [19,20]. In addition, the genes involved in synthesis of the C_30_ based carotenoids in *Bacillus firmus* [21], *Methylomonas* sp. strain 16a [13], *Planococcus maritimus* strain iso-3 [15], *Planococcus faecalis* AJ003T [22], and *P. limnophila* were also identified [17]. Notably, the genes responsible for synthesis of C_30_ based carotenoids are not always rigidly restricted in one locus [13,20,22]. Two different enzymes are characterized to catalyze the formation of the first intermediate with C_30_ backbone. The 4,4′-diapophytoene synthase CrtM and its analogues catalyze the condensation of two molecules of farnesyl diphosphate (FPP) to afford C_30_ based backbone 4,4′-diapophytoene (dehydrosqualene) or 15-*cis*-4,4′-diapophytoene [15,19]. Whereas for synthesis of C_30_ carotenoids in *P. limnophila* and *Methylobacterium extorquens* PA1, the squalene synthesized by three co-occurrence of enzymes HpnCDE is used as the first C_30_ precursor [17,23]. Next, the 4,4′-diapophytoene desaturase CrtN, 4,4′-diaponeurosporene oxidase CrtP, aldehyde dehydrogenase AldH, glycosyltransferase CrtQ, and acyltransferase CrtO are employed to introduce further tailoring modifications to diversify C_30_ carotenoids.

*Methylobacterium extorquens* AM1, one representative of methylotrophs, is a facultative methylotroph α-proteobacterium that is capable of growing in the medium with one-carbon compound as the sole carbon and energy source [24,25]. *M. extorquens* AM1 produces a carotenoid pink pigment, which is proposed to be C_30_ carotenoid rather than C_40_ carotenoid based on the following two reasons. Firstly, the pink pigment produced in *M. extorquens* PA1, the strain with closest genetic background to *M. extorquens* AM1, was shown to be C_30_ carotenoid, which is derived from squalene [23]. Secondly, phytoene synthase gene *crtB* involved in synthesis of C_40_ carotenoid in *M. extorquens* AM1 had no influence on synthesis of the pink pigment [26]. Furthermore, two desaturases META1p3665 and META1p3670 involved in synthesis of this pink pigment were identified [26,27]. Although *M. extorquens* AM1 is known to produce carotenoid for a long time, the exact structure of this carotenoid remains unknown. Herein, we report the structure of the carotenoid, as well as identification of its biosynthetic genes in *M. extorquens* AM1 by using gene deletion and gene complementation experiments.

## 2 Materials and methods

### 2.1 Strains, media and culture conditions

The plasmids and strains used and generated in this study are listed in Table 1. All *E. coli* strains were cultivated in Luria-Bertani (LB) agar or liquid medium at 37 °C supplemented with appropriate antibiotics. *M. extorquens* AM1 and its derivative strains were routinely cultivated in a minimal medium at 30 °C as described previously [31]. The final concentrations of antibiotics used in this study are 20 μg/mL tetracycline (Tet) and 25 μg/mL kanamycin (Km). All chemicals used for media were purchased from Sigma-Aldrich (St. Louis, MO, USA) unless otherwise specified.

**Table 1:** Strains and plasmids used in this study.

Plasmids/strains	Description	Sources
**Plasmids**		
pCM80	vector used for gene expression in *M. extorquens*; promoter, P_*mxaF*_; antibiotics, Tet^R^, Km^R^	[28]
pCM433	sacB-based allelic exchange vector; antibiotics, Ap^R^, Cm^R^, Tet^R^	[29]
pAIO	pCM80 derivative with *dcas9* and sgRNA expression cassette; *dcas9* and sgRNA under the control of inducible promoter P_*R/tetO*_ and P_*mxaF-g*_, respectively	[26]
pAI1815	pAIO derivative plasmid carrying the sgRNA targeting *META1p1815*	This study
pAI1816	pAIO derivative plasmid carrying the sgRNA targeting *META1p1816*	This study
pCM3663	pCM80 derivative harboring *META1p3663* under the downstream of P_*mxaF*_, Tet^R^	This study
pCM3664	pCM80 derivative harboring *META1p3664* under the downstream of P_*mxaF*_, Tet^R^	This study
pCM1815	pCM80 derivative harboring *META1p1815* under the downstream of P_*mxaF*_, Tet^R^	This study
pCM1816	pCM80 derivative harboring *META1p1816* under the downstream of P_*mxaF*_, Tet^R^	This study
pCM1815-16	pCM80 derivative harboring the operon of *META1p1815* and *META1p1816* under the downstream of P_*mxaF*_, Tet^R^	This study
pCM3665-70	pCM80 derivative harboring the operon of *META1p3665* and *META1p3670* under the downstream of P_*mxaF*_, Tet^R^	This study
pCM1815-3665-70	pCM80 derivative harboring the operon of *META1p1815, META1p3665* and *META1p3670* under the downstream of P_*mxaF*_, Tet^R^	This study
pCM-△3663	pCM433 with about 1 kb upstream and 1 kb downstream fragments of *META1p3663*; Ap^R^, Cm^R^, Tet^R^	This study
pCM-△3664	pCM433 with about 1 kb upstream and 1 kb downstream fragments of *META1p3664*; Ap^R^, Cm^R^, Tet^R^	This study
pCM-△3652	pCM433 with about 1 kb upstream and 1 kb downstream fragments of *META1p3652*; Ap^R^, Cm^R^, Tet^R^	This study
pCM-△4598	pCM433 with about 1 kb upstream and 1 kb downstream fragments of *META1p4598*; Ap^R^, Cm^R^, Tet^R^	This study
**Strains**		
*E. coli* DH5αα	Host strain for general clone	Lab storage
*M. extorquens* AM1	Wild-type, pink color, rifamycin-resistant strain	[30]
YA	*M. extorquens* AM1::pCM80	[26]
YAIO	*M. extorquens* AM1 containing the plasmid pAIO	[26]
YA3663	The gene *META1p3663* deleted from *M. extorquens* AM1	This study
YA3664	The gene *META1p3664* deleted from *M. extorquens* AM1	This study
YA3652	The gene *META1p3652* deleted from *M. extorquens* AM1	This study
YA4598	The gene *META1p4598* deleted from *M. extorquens* AM1	This study
YCM3663	The strain YA3663::pCM-exp3663, Tet^R^	This study
YCM3664	The strain YA3664::pCM-exp3664, Tet^R^	This study
YAMZ1815	*M. extorquens* AM1 harboring the CRISPRi plasmid pAI1815	This study
YAMZ1816	*M. extorquens* AM1 harboring the CRISPRi plasmid pAI1816	This study
YCM1815	*M. extorquens* AM1::pCM-exp1815, Tet^R^	This study
YCM1816	*M. extorquens* AM1::pCM-exp1816, Tet^R^	This study
YCM1815-16	*M. extorquens* AM1::pCM-exp1815-16, Tet^R^	This study
YCM3665-70	*M. extorquens* AM1::pCM-exp3665-70, Tet^R^	This study
YCM1816-3665-70	*M. extorquens* AM1:: pCM-exp1815-3665-70, Tet^R^	This study

### 2.2 Extraction and analysis of carotenoid produced by *M. extorquens* AM1 and its derivative strains

The carotenoids were extracted from *M. extorquens* AM1 as described previously [26,32]. The extract from 100 mL of cell culture was dissolved in 1 mL of CH_3_OH, and 30 μL was subject to HPLC analysis by using Waters HPLC 1260 (Waters, MA, USA) equipped with a Waters Spherisorb 5.0 μm ODS2 (4.6 mm × 250 mm, 5 μm) column [27]. The mobile phases contained solvent A (acetonitrile-water 9:1, V/V) and solvent B (methanol-isopropanol, 3:2, V/V). The elution program was set as follow: 100% A to 5% A in 0–10 min, 5% A retained from 10 to 20 min, and 5% A to 100% A in 20–25 min. The flow rate was 1.0 mL/min and UV absorbance of the peaks were detected by using a photodiode array detector.

### 2.3 Extraction and purification of carotenoid from *M. extorquens* AM1

*M. extorquens* AM1 was cultivated by using 30 L media for 5 days. Subsequently, the broth was centrifuged (8000 g for 3 min) and the pellets were extracted by using 600 mL of solvents (CH_3_OH: CHCl_3_: H_2_O = 10:3:4) until all visible pigments were removed. Next, all the bottom organic phases were combined and equal volume of acetone was added, which were incubated at 4 °C for 12 h. The insoluble pellets were removed by centrifuge and the supernatants were concentrated under reduced pressure to give the organic extract. The organic extract was fractionated by a silica gel column using gradient elution with petroleum ether-CHCl_3_-MeOH to give seven fractions (fractions 1–7). Fractions 3 and 4 eluted respectively with 98:2 petroleum ether-CHCl_3_-MeOH and 96:4 petroleum ether-CHCl_3_-MeOH were combined and separated by semi-preparative HPLC to obtain the target pigment. The elution program was identical to that used in the analysis program and the flow rate was 3.0 mL/min.

### 2.4 Gene deletion and complementation

The primers used in this study are listed in Table 2. Allelic replacement of the genes *META1p3663*, *META1p3664*, *META1p3652* and *META1p4598* in *M. extorquens* AM1 were performed by using the method described previously [26]. Briefly, about 1000 bp to 1200 bp DNA fragments located in the upstream and downstream of *META1p3663*, *META1p3664*, *META1p3652* and *META1p4598* were amplified from *M. extorquens* AM1 by PCR, respectively. Then, the two fragments of the corresponding gene were fused by overlapping PCR. The PCR products with correct sizes were purified and ligated into linear pCM433 vector digested by *Bgl* II and *Sac* I through recombinant clone strategy, respectively. The transformants were screened by PCR and the positive clones were verified by sequencing, the correct plasmids were termed as pCM-△3663, pCM-△3664, pCM-△3652 and pCM-△4598. Subsequently, the plasmids pCM-△3663, pCM-△3664, pCM-△3652 and pCM-△4598 were electroporated into *M. extorquens* AM1, respectively. *M. extorquens* AM1 bearing the corresponding plasmid was firstly selected by using tetracycline, then, the double-crossover mutants occurred by growing on the plates containing 5% sucrose (w/v). The genotype of mutant strains with successful allele swapping were firstly screened by PCR, followed by sequencing of the PCR fragments with expected size. The correct mutant strains were termed as YA3663, YA3664, YA3652 and YA4598.

**Table 2:** Primers and sgRNAs used in this study.

Primers	Sequences (5′-3′)	Usage
3663Up-F61	GCCACCTGACGTCTAGATCTCGAGCAGATCCTCGATCGCG	Construction of pCM-△3663 and PCR verification of YA3663
3663Up-R58	TCCGAGGAGACGCGGACCCGGGGACTTTTCGCGAAACAATCC	Construction of pCM-△3663
3663Down-F63	ATTGTTTCGCGAAAAGTCCCCGGGTCCGCGTCTCCTCG	Construction of pCM-△3663
3663Down-R61	CTGGATCCTCTAGTGAGCTCCGCGATCTACGGGCTGGC	Construction of pCM-△3663 and PCR verification of YA3663
3664Up-F59	GCCACCTGACGTCTAGATCTGAGAATCGTCGCGAACGGC	Construction of pCM-△3664 and PCR verification of YA3664
3664Up-R60	ACCGCCTGAGTTGATTCGACCACTCACCCTCCTCGTCCTC	Construction of pCM-△3664
3664Down-F58	GAGGACGAGGAGGGTGAGTGGTCGAATCAACTCAGGCGGT	Construction of pCM-△3664
3664Down-R60	CGCTCGAGCTGCAGCATATGGGCAAGGTCAAGGATGCGC	Construction of pCM-△3664 and PCR verification of YA3664
3652up-F58	GCCACCTGACGTCTAGATCTCCGGCTTCCTCTCGATCAAC	Construction of pCM-△3652
3652up-R62	GTCTCGAACTCTCGAAGGTCCGCGTTTCCTCCATTGCGCT	Construction of pCM-△3652
3652down-F55	AGCGCAATGGAGGAAACGCGGACCTTCGAGAGTTCGAGAC	Construction of pCM-△3652
3652down-R57	CTGGATCCTCTAGTGAGCTCTGCTCACCTCGAAGTTCAGA	Construction of pCM-△3652
3652-tF	AGATCCGCTTCACCGCCGA	PCR verification of strain YA3652
3652-tR	TGACAGGGACGGGCTGAAGC	PCR verification of strain YA3652
4598-Up-F	AAAGTGCCACCTGACGTCTAGATCTACGAGATGGTCGAACATCGCGT	Construction of pCM-△4598
4598-Up-R	TGCCTATATGAAGGCCATCCCTCGTATCACCGGTCAAGCGTGTCTCGAA	Construction of pCM-△4598
4598-Down-F	TTCTTCGAGACACGCTTGACCGGTGATACGAGGGATGGCCTTCATATA	Construction of pCM-△4598
4598-Down-R	TCGGCTGGATCCTCTAGTGAGCTCTCGATACCGCCTCGACCTATT	Construction of pCM-△4598
4598-tF	GGGATTGAACGGGTTTTCGC	PCR verification of YA4598
4598-tR	GGTGCCCGTGATGTGCCTGA	PCR verification of YA4598
Com-R	CTATATTTTCTAGGCTTTGATTG	Construction of plasmid pAI1815 and pAI1816
1815-NT766-F	TCAAAGCCTAGAAAATATAGTGATGGACTTTCTCGCTGAGGTTTTAGAGCTAGAAATAG	Construction of plasmid pAI1815
1816-NT802-F	TCAAAGCCTAGAAAATATAGAGGAGAAGGGGGAATTTGCCGTTTTAGAGCTAGAAATAG	Construction of plasmid pAI1816
80-15F63	ACCATGATTACGCCAAGCTTATGAGCGCCGCGCTTCAAAC	Construction of pCM-exp1815 and pCM-exp1815-1816
80-15R70	ACGGGATTCTGTGAGGATCCTCATCGGCCGGCTCCGGCGG	Construction of pCM-exp1815
80-16F69	ACCATGATTACGCCAAGCTTATGAGCGCCACCGCCACCCC	Construction of pCM-exp1816
80-16R63	AGCTCGGTACCCGGGGATCCTCACAGAATCCCGTGGCGCA	Construction of pCM-exp1816 and pCM-exp1815-1816
80-3663F-57	ACCATGATTACGCCAAGCTTATGACACTCACCCTCCTCG	Construction of pCM-exp3663
80-3663R-58	AGCTCGGTACCCGGGGATCCTCAACCCGGTTCCTGCC	Construction of pCM-exp3663
80-3664-F60	ACCATGATTACGCCAAGCTTATGGCGCGGGGCAAAC	Construction of pCM-exp3664
80-3664-R60	AGCTCGGTACCCGGGGATCCTCATCGGGTCCGCGTCTC	Construction of pCM-exp3664

For the mutant strains YA3663 and YA3664, the gene complementation experiments were performed as described previously [26]. The genes *META1p3663* and *META1p3664* were amplified from *M. extorquens* AM1 by using PCR, and then cloned into vector pCM80 under downstream of promoter P_*mxaF*_ to afford the plasmids pCM-exp3663 and pCM-exp3664, respectively. After confirming the inserted fragments by sequencing, pCM-exp3663 and pCM-exp3664 were introduced into corresponding mutant strains by using electroporation to afford the strains YCM3663 and YCM3664, respectively.

### 2.5 CRISPR interfering of *META1p1815 and META1p1816*

By using reverse PCR technology with plasmid pAIO as template [26], two plasmids pAI1815 (targeting *METAp1815*) and pAI1816 (targeting *METAp1816*) were generated, which were introduced into *M. extorquens* AM1 to afford the strains YAMZ1815 and YAMZ1816, respectively.

### 2.6 Gene overexpression in *M. extorquens* AM1

The gene overexpression strains YCM1815, YCM1816 and YCM1815-16 were generated as below. The individual genes *META1p1815, META1p1816* and the operon *META1p1815-META1p1816* were amplified from *M. extorquens* AM1 by using PCR, respectively. Then, the PCR fragments with expected size were purified and then ligated into vector pCM80 under downstream of promoter P_maxF_, generating plasmids pCM1815, pCM1816 and pCM1815-16, respectively. After verifying by sequencing, pCM1815, pCM1816 and pCM1815-16 were introduced into *M. extorquens* AM1 by electroporation to afford YCM1815, YCM1816 and YCM1815-16, respectively.

The strain YCM3665-70 was generated as below. The individual genes *META1p3665* and *META1p3670* were amplified by using PCR. Then, the two fragments with correct size were purified, which then were fused by overlapping PCR. Next, the fused *META1p3665* and *META1p3670* fragment was cloned into pCM80 vector to afford plasmid pCM3665-70. After confirming by sequencing, the plasmid pCM3665-70 was electroporated into *M. extorquens* AM1 to generate YCM3665-70.

The construction of YCM1815-3665-70 was described as below. Briefly, the fragments of *META1p1815* and *META1p3665*-*META1p3670* were fused by overlapping PCR to generate *META1p1815*-*META1p3665*-*META1p3670* cassette, which was then cloned into pCM80 vector to afford the plasmid pCM1815-3665-70. After confirming by sequencing, pCM1815-3665-70 was introduced into *M. extorquens* AM1 by using electroporation, leading to generate YCM1815-3665-70.

### 2.7 Spectroscopic analyses of carotenoids from *M. extorquens* AM1 and its derivative strains

^1^H NMR spectrum was recorded at 25 °C on Bruker AV 500 instruments. LC-HR-MS data were acquired on a Thermo MAT95XP high-resolution mass spectrometer or a Waters micro MS Q-Tof spectrometer.

## 3 Results

### 3.1 Isolation and structural determination of the C_30_ carotenoid pigment

**Fig. 1:**
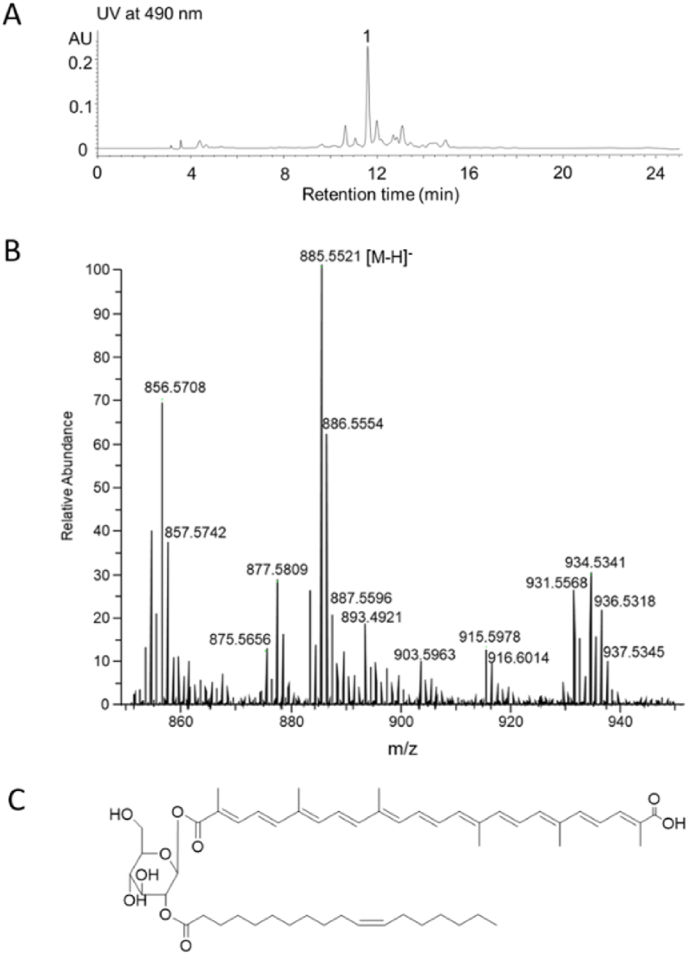
HPLC analysis (A), mass spectrophotometry (MS) analysis (B), and structure (C) of the carotenoids extracted from *M. extorquens* AM1. (A) The HPLC profile of the metabolites produced by *M. extorquens* AM1. The compound **1** with retention time at 11.6 min was isolated for further NMR analyses; (B) The mass of compound **1**, [M − H]^-^ = 885.5521 (observed), [M − H]^-^ = 885.5517 (calculated); (C) the structure of compound **1**.

The carotenoid pigments extracted from *M. extorquens* AM1 were analyzed by HPLC. The major peak **1** at 11.6 min with characteristic absorption at 490 nm as well as several trace congers were detected (Fig. 1A). Next, LC-MS analysis revealed that peak **1** has a molecular mass of 886.5521 (detected as [M − H]^-^ = 885.5521, Fig. 1B), implying that its molecular formula is C_54_H_78_O_10_ (calculated [M − H]^-^ = 885.5517). By searching the carotenoids produced by *Methylorubrum* species, we found that compound **1** has the same molecular mass and molecular formula to 4-[2-*O*-11*Z*-octadecenoyl-β-glucopyranosyl]-4,4′-diapolycopenedioc acid, a glycosylated carotenoid produced by *M. populi* BJ001 [14], suggesting they shared the same chemical structure. Further analysis of ^1^H NMR data proved our speculation, as their hydrogen signals were almost identical (Fig. S1). Therefore, the compound **1** was proposed to be 4-[2-*O*-11*Z*-octadecenoyl-β-glucopyranosyl]-4,4′-diapolycopenedioc acid.

### 3.2 Three enzymes META1p1815, META1p1816 and META1p1817 participate in synthesis of precursor squalene

By using diapophytoene synthase CrtM as a probe, two analogous proteins, presqualene diphosphate synthase META1p1816 (HpnD, 28.5% identity to CrtM) and META1p3220 (CrtB, 27.7% identity to CrtM) were identified. Previous studies demonstrated that the phytoene synthase CrtB is not related to synthesize carotenoids in *M. extorquens* AM1 and *M. extorquens* PA1 [23,27], which suggest META1p1816 is probably the candidate protein. Further analyses of *M. extorquens* AM1 genome revealed that four genes encoding META1p1815 (HpnC), META1p1816 (HpnD), META1p1817 (HpnE), and squalene-hopene cyclase META1p1818 (HpnF/SchC) were clustered in one locus. The three co-occurrence of enzymes HpnCDE were confirmed to synthesize precursor squalene in C_30_ carotenoid pathway in *M. extorquens* PA1 and *P. limnophila* [17,23]. Given META1p1815, META1p1816, META1p1817 show nearly 100% identities to their counterpart proteins HpnCDE presented in *M. extorquens* PA1, we therefore propose that META1p1815, META1p1816, META1p1817 are used to synthesize C_30_ precursor squalene in compound **1** biosynthetic pathway in *M. extorquens* AM1.

The pink carotenoid pigment and hopanoid share the same biosynthetic intermediate in *M. extorquens* AM1 [23,26]. Hopanoid plays essential roles in physiological processes such as membrane fluidity and lipid packing in *M. extorquens*, thus, deletion of gene *shc* encoding squalene-hopene cyclase led the mutant strain has a bad growth [23,32]. To avoid impairment on the growth of *M. extorquens* AM1 caused by deleting genes related to synthesis of precursor squalene, we used CRISPR interfering technology to decrease the expression of *META1p1815* and *META1p1816*. No matter of interfering *META1p1815* and *META1p1816*, the growth of *M. extorquens* AM1 was significantly attenuated (Table S1).

**Fig. 2:**
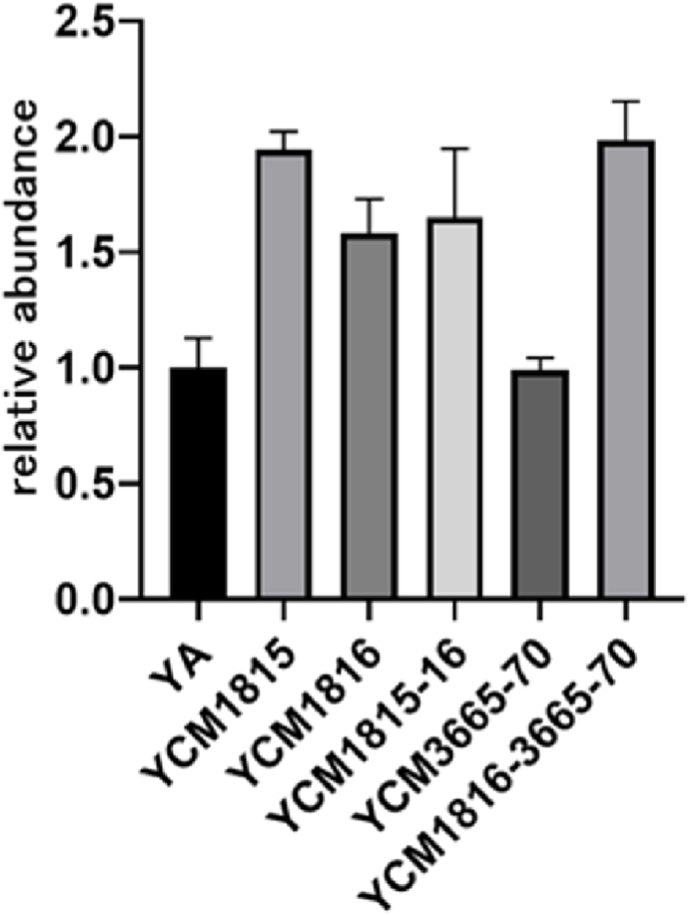
The relative titer of **1** in *M. extorquens* AM1 derivative strains. YA is *M. extorquens* AM1 harboring empty pCM80, which is used as control strain. YCM1815, YCM1816, and YCM1815-16 are *M. extorquens* AM1 derivative strains which carry plasmids for overexpressing the genes *META1p1815*, *META1p1816*, *META1p1815* and *META1p1816*, respectively. YCM3665-70 is *M. extorquens* AM1 derivative strain which carries plasmid for overexpressing genes *META1p3665* and *META1p3670*. YCM1816-3665-70 is *M. extorquens* AM1 derivative strain which carries the plasmid for overexpressing the cassette of *META1p1816*, *META1p3665* and *META1p3670*.

Next, the involvement of META1p1815 (HpnC) and META1p1816 (HpnD) in biosynthetic pathway of compound **1** was investigated by gene overexpression experiments. Compared to *M. extorquens* YA harboring empty pCM80, the titer of **1** in YCM1815 (overexpression of *META1p1815*), YCM1816 (overexpression of *META1p1816*), and YCM1815-16 (overexpression of *META1p1815* and *META1p1816* cassette) increased by 94.4%, 58.2%, and 65.1%, respectively (Fig. 2), suggesting that increasing the precursor supply improves the titer of compound **1**. As revealed from the titer of **1** in YCM3665-70, overexpression of genes encoding enzymes related to modify precursor squalene cannot improve the titer of **1** (Fig. 2). However, co-expression of genes involved in precursor squalene synthesis and modification can significantly improve the titer of **1**, as revealed from the titer of **1** in YCM1816-3665-70 (Fig. 2). These results suggested that the supply of the precursor squalene maybe the bottleneck of the titer of carotenoid **1**.

### 3.3 Mining the oxidases in synthesis of compound 1

After formation of squalene, multiple oxidative modifications are required to afford the intermediate 4,4′-diapolycopene-4,4′-dioic acid [20]. META1p3670 and META1p3665 were confirmed in synthesizing the C_30_ carotenoid in *M. extorquens* AM1 [26,27], however, the exact roles of META1p3670 and META1p3665 are still not well characterized. META1p3670 shows 30.4% identity to CrtN involved in staphyloxanthin biosynthetic pathway. Since its counterpart WP_012254689.1 (Mext_3436) presented in *M. extorquens* PA1 (100% identity to META1p3670) is classified as CrtN group protein by using phylogenetically analysis [23], therefore, META1p3670 is proposed to act as a desaturase to catalyze multiple desaturation steps. META1p3665 shows 32.5% identity to CrtP involved in staphyloxanthin biosynthetic pathway, and its counterpart protein WP_003603441.1 (100% identity to META1p3665) in *M. extorquens* PA1 acts as a CrtP-type oxidase [23], therefore, META1p3665 is proposed to act as an oxidase to catalyze the formation of terminal aldehyde groups.

AldH was reported to catalyze the formation of terminal carboxylic acid in staphyloxanthin biosynthetic pathway [20]. Then, by using AldH as a probe, three homologous proteins, the aldehyde dehydrogenase META1p3652 (29.6% identity to AldH), the succinate-semialdehyde dehydrogenase I META1p4598 (25.8% identity to AldH), and the proline dehydrogenase META1p0211 (27.2% identity to AldH) were found in *M. extorquens* AM1 genome. META1p3652 (507 amino acids, a.a.) and META1p4598 (477 a.a.) have the similar amino acid numbers to AldH (459 a.a.), whereas the protein length of META1p0211 (1035 a.a.) is much longer than that of META1p3652 and META1p4598. Thus, the candidates of AldH in *M. extorquens* AM1 are more likely to be META1p3652 and META1p4598.

Subsequently, the roles of *META1p3652* and *META1p4598* in compound **1** biosynthetic pathway were investigated by using gene deletion experiments. Unfortunately, both mutant strains YA3652 (Δ*META1p3652*) and YA4598 (Δ*META1p3652*) still produce carotenoid **1** (Fig. S2), suggesting both META1p3652 and META1p4598 are not involved in synthesis of **1.**

### 3.4 META1p3663 acts as a glycosyltransferase in compound 1 biosynthetic pathway

**Fig. 3:**
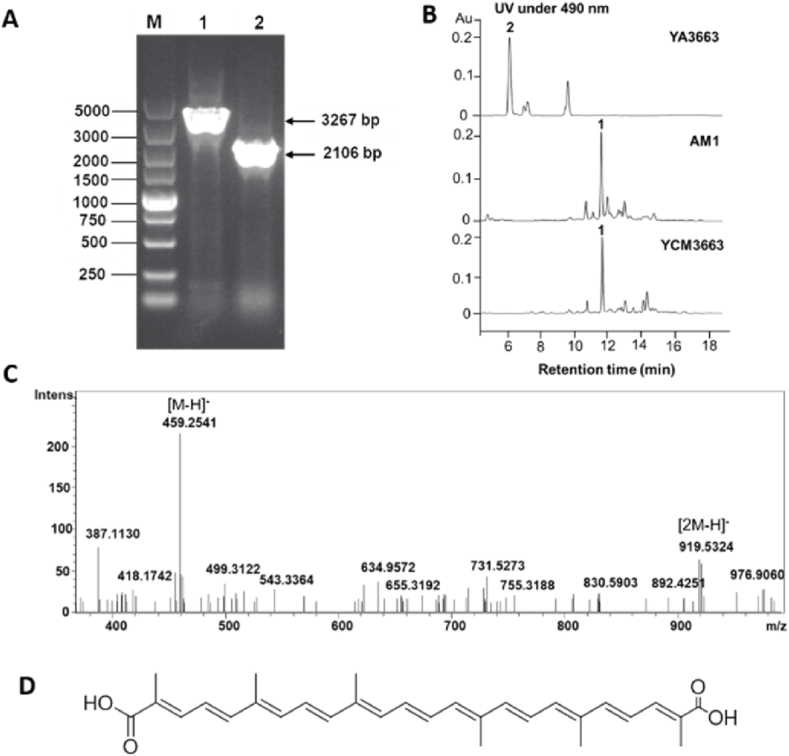
Generating *META1p3663* deletion mutant strain YA3663 and analyzing its metabolites. (A) Verification of genotype of YA3663 by using PCR, lane M: DNA ladder marker, lane 1: PCR fragments (2106 bp) from *M. extorquens* AM1 wild type, lane 2: PCR fragments (3267 bp) from mutant strain YA3663. (B) HPLC analyses of metabolites from YA3663, *M. extorquens* AM1 wild type, and YCM3663. (C) LC-HR-MS analyses of compound 2 generated by strain YA3663. The observed mass of compound **2** is [M − H]^-^ = 459.2541, [2M − H]^-^ = 919.5324, the calculated mass is [M − H]^-^ = 459.2535. (D) The proposed structure of compound **2** based on mass and biosynthetic pathway.

The subsequent modification of 4,4′-diapolycopene-4,4′-dioic acid is glycosylation. In *M. extorquens* AM1, the glycosyltransferase META1p3663, displaying 27.32% identity to CrtQ, is found to be candidate to catalyze the glycosylation reaction, leading to afford glucosyl-4,4′-diapolycopene-4,4′-dioic acid. To verify the role of glycosyltransferase META1p3663 in compound **1** biosynthetic pathway, *META1p3663* was deleted from *M. extorquens* AM1 genome, generating the mutant strain YA3663 (Fig. 3). The strain YA3663 still shows light pink color, implying that the carotenoid pigment is still produced in this strain. The light pink pigment extracted from YA3663 was analyzed by HPLC. Compared to *M. extorquens* AM1 wild type, YA3663 abolished the production of compound **1**, instead, two new peaks (the main product **2** at 6.02 min and one minor peak at 9.5 min) with the characteristic ultraviolet absorption of carotenoid were detected. Then, molecular formula of the compound **2** was determined as C_30_H_36_O_4_ based on LC-HR-MS data (observed mass [M − H]^-^ = 459.2536, [2M − H]^-^ = 919.5324, calculated mass [M − H]^-^ = 459.2535), which was identical to the expected intermediate 4,4′-diapolycopene-4,4′-dioic acid (Fig. 3). Furthermore, to evaluate whether the production of 4,4′-diapolycopene-4,4′-dioic acid was caused by deletion of *META1p3663*, the gene *META1p3663* was re-introduced into YA3663 to afford strain YCM3663. HPLC analyses results revealed that YCM3663 strain restores the production of compound **1**, suggesting the glycosyltransferase META1p3663 is responsible for the glycosylation process.

### 3.5 META1p3664 acts as an acyltransferase in compound 1 biosynthetic pathway

The last tailoring step in compound **1** biosynthetic pathway is acylation of glucosyl-4,4′-diapolycopene-4,4′-dioic acid. When the acyltransferase CrtO was used as a probe, no homologous proteins were found in *M. extorquens* AM1 genome. Further bioinformatics analyses demonstrated that a lysophospholipid acyltransferase META1p3664 located next to the glycosyltransferase META1p3663. Because of the co-occurrence of META1p3664 and META1p3663, META1p3664 is probably the candidate enzyme to catalyze the last step in compound **1** biosynthetic pathway.

**Fig. 4:**
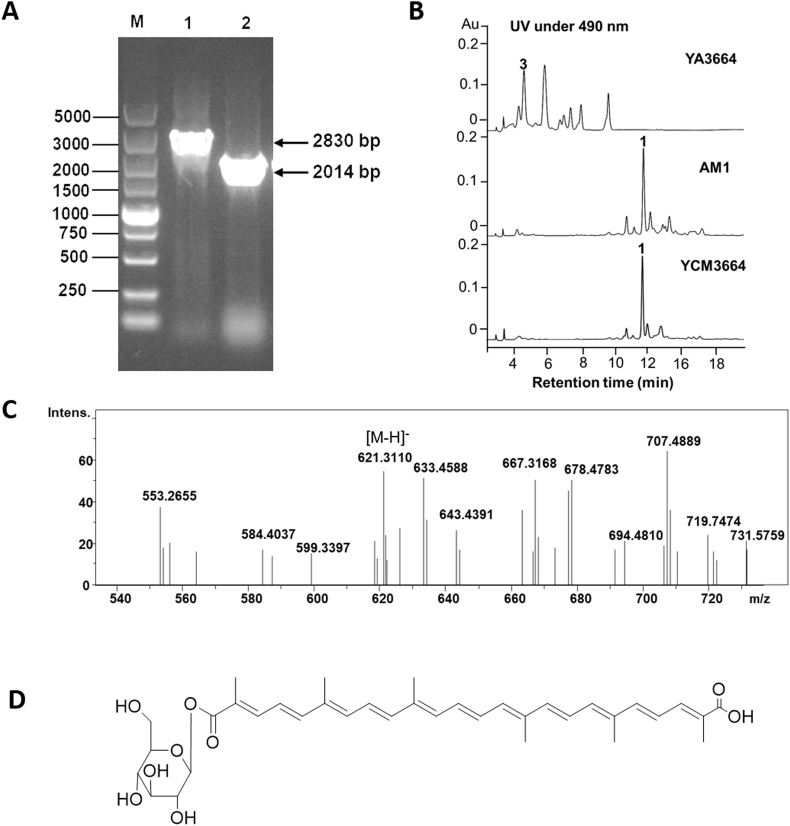
Generating *META1p3664* deletion mutant strain YA3664 and analyzing its metabolites. (A) Verification of the genotype of the mutant strain YA3664 by using PCR, lane M: DNA ladder marker, lane 1: PCR fragments (2830 bp) from *M. extorquens* AM1 wild type, lane 2: PCR fragments (2014 bp) from mutant strain YA3664. (B) HPLC analyses of the metabolites from YA3664, *M. extorquens* AM1 wild type, and YCM3664 (YA3664 complemented with *META1p3664*). (C) LC-HR-MS analyses of compound **3** generated by mutant strain YA3664. The observed mass of compound **3** is [M − H]^-^ = 621.3110, the calculated mass is [M − H]^-^ = 621.3064. (D) The proposed structure of compound **3** based on mass and biosynthetic pathway analyses.

To check the role of META1p3664 in synthesis of compound **1**, *META1p3664* was deleted from the genome to afford the strain YA3664 (Fig. 4). The strain YA3664 still shows light pink color. By comparison of the wild type *M. extorquens* AM1, YA3664 lost the capacity to produce carotenoid **1**, instead, several peaks with characteristic ultraviolet absorption of carotenoid at 490 nm were detected (Fig. 4). To check whether the production of these carotenoid derivatives in YA3664 was caused by deletion of *META1p3664*, the gene *META1p3664* was re-introduced into YA3664. The strain YCM3664 harboring *META1p3664* driven by promoter p_*mxaF*_ in pCM80 plasmid can restore the production of compound **1**, thus, META1p3664 was confirmed to be involved in synthesis of compound **1**. Then, the metabolites produced in YA3664 were analyzed by LC-HR-MS. The compound **3** with target molecular mass was observed ([M − H]^-^ = 621.3110), which is in accordance with the predicted intermediate glucosyl-4,4′-diapolycopene-4,4′-dioic acid (calculated mass [M − H]^-^ = 621.3064) (Fig. 4). Based on these results, META1p3664 was confirmed to catalyze the last acylation reaction to afford compound **1**.

## 4 Discussion and conclusion

Although *M. extorquens* AM1 is known to produce pigment for a long time, the structure of the carotenoid remains unknown. Herein, we firstly purified the carotenoid and determined its structure as 4-[2-*O*-11*Z*-octadecenoyl-β-glucopyranosyl]-4,4′-diapolycopenedioc acid, a glycosylated carotenoid with C_30_ backbone. Furthermore, two enzymes, the glycosyltransferase META1p3663 and lysophospholipid acyltransferase META1p3664, were identified to participate in synthesis of the C_30_ carotenoid.

*Methylobacterium* species, a representative group of methylotroph strains widely distributed in phyllosphere region of plants, are found to be good candidates as plant growth-promoting bacteria because they can provide nutrients to plants, modulate phytohormone levels, and protect plants against pathogens [33,34]. To resist harmful UV radiations during phyllosphere colonization and/or used for anoxygenic photosynthesis, *Methylobacterium* species often synthesize carotenoids to provide natural antioxidant activity [14,33,35]. Up to now, only C_30_ carotenoids were reported from *Methylobacterium* species such as *M. populi* BJ001, *M. radiotolerans* JCM2831 and *M. rhodinum* ATCC 14821 [14,36]. Genome analyses revealed that the key enzyme CrtM in C_40_ carotenoid synthesis via CrtB-CrtI-CrtD pathway is absent in alpha-proteobacteria [23], therefore, the *Methylobacterium* species belonging to alpha-proteobacteria cannot produce C_40_ carotenoid. Some C_30_ carotenoids possess better antioxidant activity in both the physical and chemical quenching of reactive oxygen species [37]. The compound **1** isolated here was reported to show better antioxidative activity than C_40_ carotenoid astaxanthin by using singlet oxygen quenching model experiment [14]. Some other C_30_ carotenoids such as 4,4′-diapolycopene-4,4′-dial, methyl glucosyl-3,4-dehydroapo-8′-lycopenoate also displayed better antioxidant activity than C_40_ carotenoids [14,38,39]. These C_30_ carotenoids are proposed to protect *Methylobacterium* species against photosensitization reactions because they grow on plant leaves where they are exposed to strong sunlight [14]. It was shown that disruption of C_30_ carotenoid synthesis led slightly increasing sensitivity of *M. extorquens* to oxidative stress [23].

**Fig. 5:**
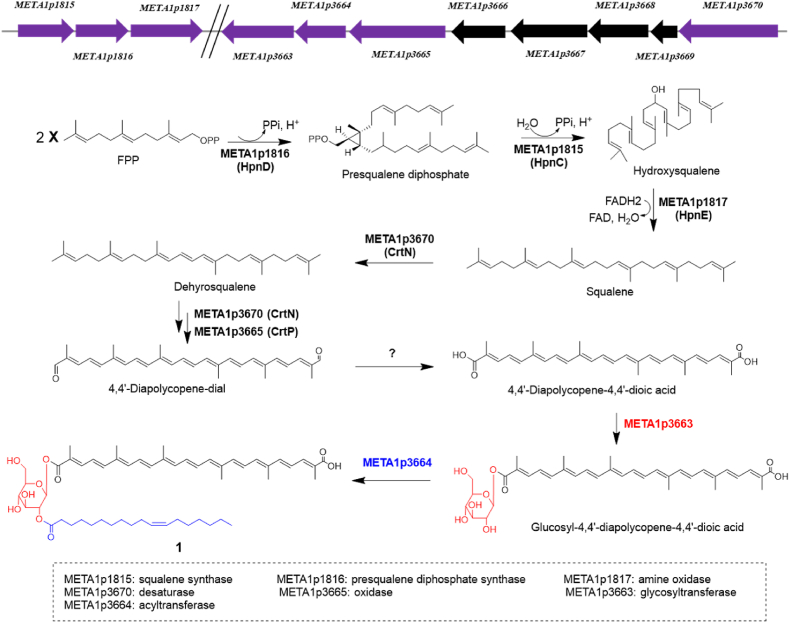
The gene organization and biosynthetic pathway of compound **1**. The arrows in pink represent the genes involved in biosynthesis of compound **1**.

As for the first C_30_ intermediate in C_30_ carotenoid biosynthetic pathway, two different routes were characterized. The first route is diapophytoene synthase CrtM catalyzes the condensation of two molecules of FPP to afford the C_30_ intermediate 4,4′-diapophytoene (staphyloxanthin biosynthetic pathway in *Staphylococcus aureus*) or 15-*cis*-4,4′-diapophytoene (methyl 5-glucosyl-5,6-dihydro-4,4′-diapolycopenoate biosynthetic pathway in marine bacterium *Planococcus maritimus* strain iso-3) [15,20]. The secondary route is that squalene synthesized by squalene synthase or three co-occurrence of enzymes HpnCDE is used as precursor [17,23]. Bioinformatics analyses demonstrated that squalene used as precursor in C_30_ carotenoid biosynthesis via HpnCDE pathway is widespread in prokaryotes [17,23]. In *M. extorquens* PA1 and *P. limnophila*, this squalene route to C_30_ carotenoids is confirmed by gene knockout or gene heterologous expression experiments [17,23]. In this study, the three enzymes META1p1815 (HpnC), META1p1816 (HpnD) and META1p1817 (HpnE) were assumed to synthesize the precursor squalene (Fig. 5), further gene overexpression experiments also confirmed their participation in synthesis of the C_30_ carotenoid.

The tailoring steps from squalene to 4,4′-diapolycopene-4,4′-dioic acid in *M. extorquens* AM1 were not well characterized. Based on phylogenetically analyses, META1p3670 and META1p3665 were classified as desaturase (CrtN) and oxidase (CrtP), respectively. However, the exact tailoring timing of META1p3670 and META1p3665 remains elusive. Previous studies revealed that Δ*META1p3665* and Δ*META1p3670* strains are colorless [26,27]. Likewise, deletion of *WP_012254689.1* (*crtN*) or *WP_003603441.1* (*crtP*) also lead to abolish the production of carotenoid in *M. extorquens* PA1, moreover, the precursors for carotenoid synthesis were not detectable in both mutant strains [23]. The colorless phenotype of the mutant strains (*ΔMETA1p3665* and *ΔWP_003603441.1*) demonstrated that the native substrate of META1p3665 or WP_003603441.1 is not 4,4′-diapolycopene, otherwise, the mutant strains can still present pink color for the accumulation of 4,4′-diapolycopene. These results give us a hint that the substrate of META1p3665 should be an intermediate in biosynthesis of 4,4′-diapolycopene rather than 4,4′-diapolycopene. As for the exact timing of terminal aldehyde group catalyzed by META1p3665, it remains unknown (Fig. 5). The aldehyde dehydrogenases such as AldH catalyzing the transformation from 4,4′-diapolycopene-dial to 4,4′-diapolycopene-4,4′-dioic acid are often not clustered with other biosynthetic genes [13,20]. In this study, two AldH analogues META1p3652 and META1p4859, albeit the identities to AldH are low, showed no influence on production of carotenoid **1**. This result suggests that the missing aldehyde dehydrogenase in *M. extorquens* AM1 maybe different from AldH and its analogues, which requires further investigations. After formation of 4,4′-diapolycopene-4,4′-dioic acid, META1p3663 and META1p3664 catalyze the glycosylation and acylation modification of 4,4′-diapolycopene-4,4′-dioic acid to afford compound **1** (Fig. 5).

**Fig. 6:**
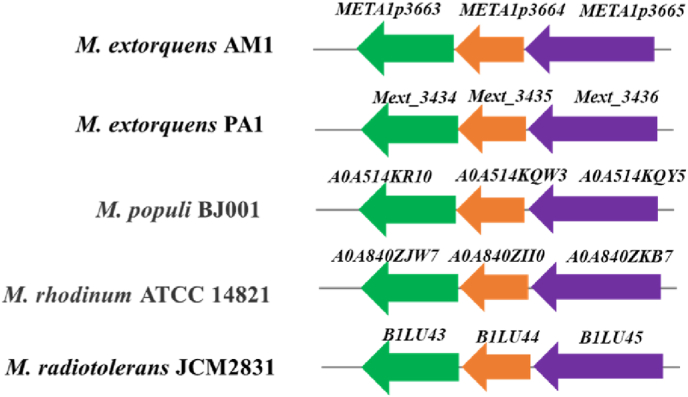
The gene cassettes containing *META1p3663* (encoding glycosyltransferase), *META1p3664* (acyl transferase) and *META1p3665* (*crtP*) were identified from *Methylobacterium* strains which produce C_30_ carotenoids with characterized structures.

Some new peaks harboring almost identical UV–visible spectra to the C_30_ carotenoid were detected in mutant strains YA3664 and YA3663, but further LC-HR-MS analyses revealed that they were not related to the intermediates in compound **1** biosynthetic pathway. Given the terminal carboxylic acid is prone to be esterified, we hypothesize that these peaks are esterified derivatives, and this phenomenon was also observed in *Planctopirus limnophila* and *M. extorquens* PA1 [[17], [23], [40], [41]]. Bioinformatics analyses revealed that the three genes cassette containing *META1p3663*, *META1p3664* and *META1p3665* located in the genomes of *Methylobacterium* strains *M. extorquens* PA1, *M. populi* BJ001, *M. radiotolerans* JCM2831 and *M. rhodinum* ATCC 14821 (Fig. 6). *M. populi* BJ001, *M. radiotolerans* JCM2831 and *M. rhodinum* ATCC 14821 were found to produce glycosylated C_30_ carotenoid, which were consistent with the biosynthetic genes in these strains. Notably, the C_30_ carotenoids produced by *M. extorquens* PA1 are different from *M. extorquens* AM1 [23]. Based on the gene structure analyses, *Mext_3434*, *Mext_3435* and *Mext_3436* (*crtP*) probably are transcribed as one operon, in which *Mext_3436* is proven to be active [23]. However, Mext_3434 and Mext_3435 show no modification toward 4,4′-diapolycopene-4,4′-dioic acid. One possible reason is that an unknown methyltransferase in *M. extorquens* PA1 is more active than the glycosyltransferase *Mext_3434*, which can easily methylate the terminal carboxylic acid at 4,4′-diapolycopene-4,4′-dioic acid to form ester bond, therefore, further glycosylation cannot proceed.

In conclusion, the C_30_ carotenoid 4-[2-*O*-11*Z*-octadecenoyl-β-glucopyranosyl]-4,4′-diapolycopenedioc acid was firstly identified from *M. extorquens* AM1. By combination of bioinformatics analyses and gene deletion experiments, the genes involved in synthesis of compound **1** were identified. The genes related to synthesizing C_30_ carotenoid are not rigidly clustered in one locus of *M. extorquens* AM1 genome. At last, the function of two new enzymes glycosyltransferase META1p3663 and lysophospholipid acyltransferase META1p3664 in C_30_ carotenoid biosynthetic pathway were characterized by using gene deletion and gene complementation experiments.

## CRediT authorship contribution statement

**Xu-Hua Mo:** Designed the project, Formal analysis, analyzed the data, Writing – original draft, wrote original draft. **Yu-Man Sun:** Carried out the experiments, Formal analysis, analyzing the data. **Yu-Xing Bi:** Carried out the experiments, Formal analysis, analyzed the data. **Yan Zhao:** Carried out the experiments. **Gui-Hong Yu:** Analyzed the data. **Ling-ling Tan:** Supervision, Designed and supervised the project, Revised the manuscript. **Song Yang:** Supervision, Designed and supervised the project, Revised the manuscript.

## Declaration of competing interest

The paper entitled “Characterization of C30 carotenoid and identification of its biosynthetic gene cluster in *Methylobacterium extorquens* AM1” was submitted to Synthetic and Systems Biotechnology. All authors declare that they do not have any financial or commercial conflict of interest in connection with the work submitted.
