# Supplementary material for: Characterization of C30 carotenoid and identification of its biosynthetic gene cluster in Methylobacterium extorquens AM1
Source: Synth Syst Biotechnol. 2023 Aug 9;8(3):527–35. doi: 10.1016/j.synbio.2023.08.002 (PMC10448405; doi:10.1016/j.synbio.2023.08.002)
Supplement: Multimedia component 1 [file mmc1.docx]

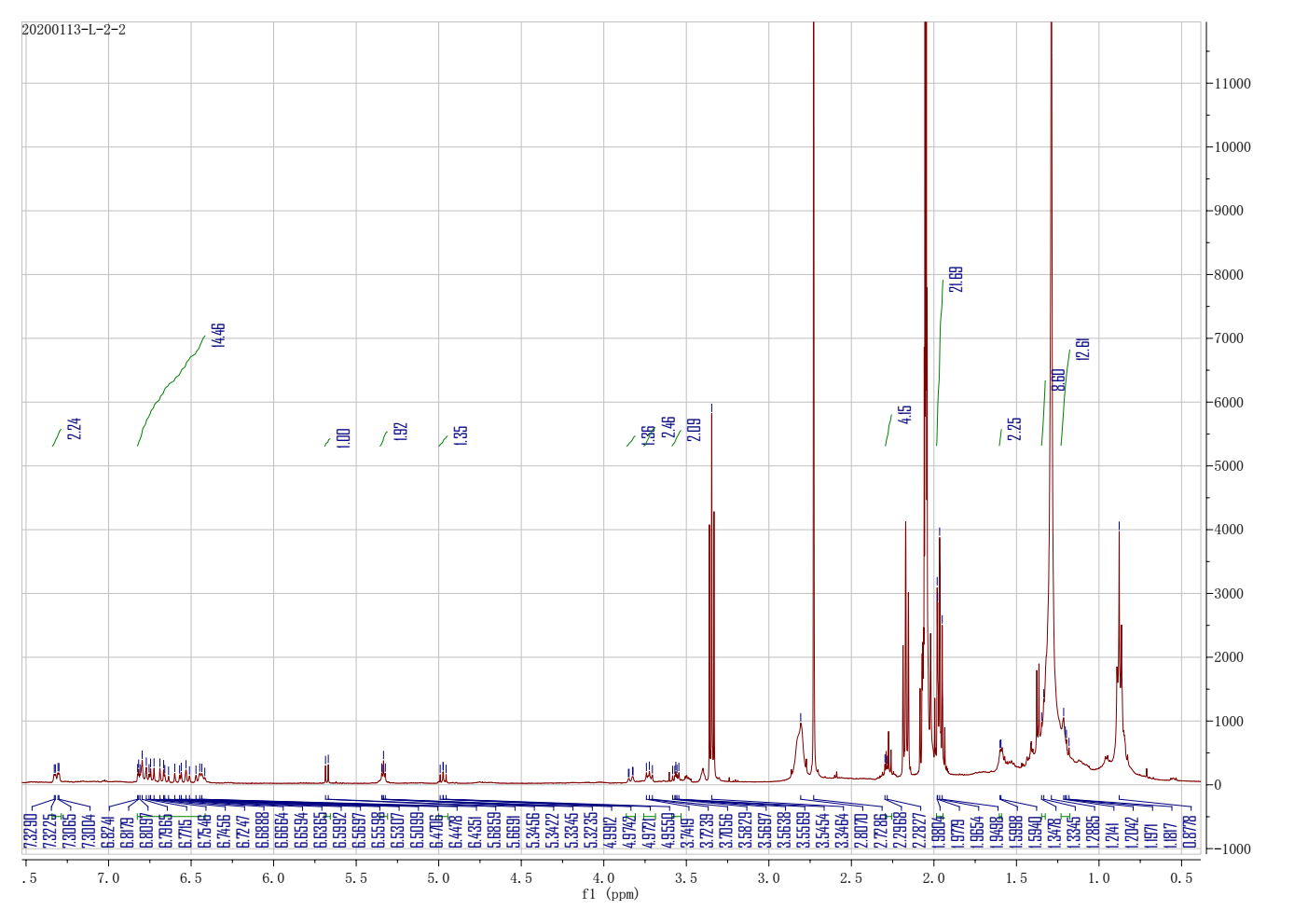


^1^H NMR [500 MHz, (CD_3_)_2_CO] *δ*: 7.32 (d, 1H, *J* = 11.3 Hz, H-6), 7.31 (d, 1H, *J* = 11.1 Hz,H-6'), 6.41-6.83 (overlap, 14H, H-7, H-7', H-8, H-8', H-10, H-10', H-11, H-11', H-12, H-12', H-14, H-14', H-15, H-15'), 5.68 (d, 1H, *J* = 8.4 Hz, H-1''), 5.34 (m, 2H, H-11''', H-12'''), 4.97 (dd, *J* = 9.6, 8.5 Hz, H-2''), 3.84 (dd, 1H, *J* = 12.2, 1.9 Hz, H-6''), 3.75 – 3.70 (overlap, 2H, H-6'', H-3''), 3.58 – 3.54 (overlap, 2H, H-5'', H-4''), 2.28 (overlap, 2H, H_2_-2'''), 2.00 – 1.94 (overlap, 21H, H_2_-10''', H_2_-13''', H_3_-18' – H_3_-20', H_3_-18 – H_3_-20), 1.60 (m, 2H, H-3'''), 1.33 – 1.20 (m, 20H, H-4''' – H-9''', H-14''' – H-17'''), 0.88 (overlap, 3H, H-18''').

Figure S1. The ^1^H NMR spectrum and data of the carotenoid compound 1.


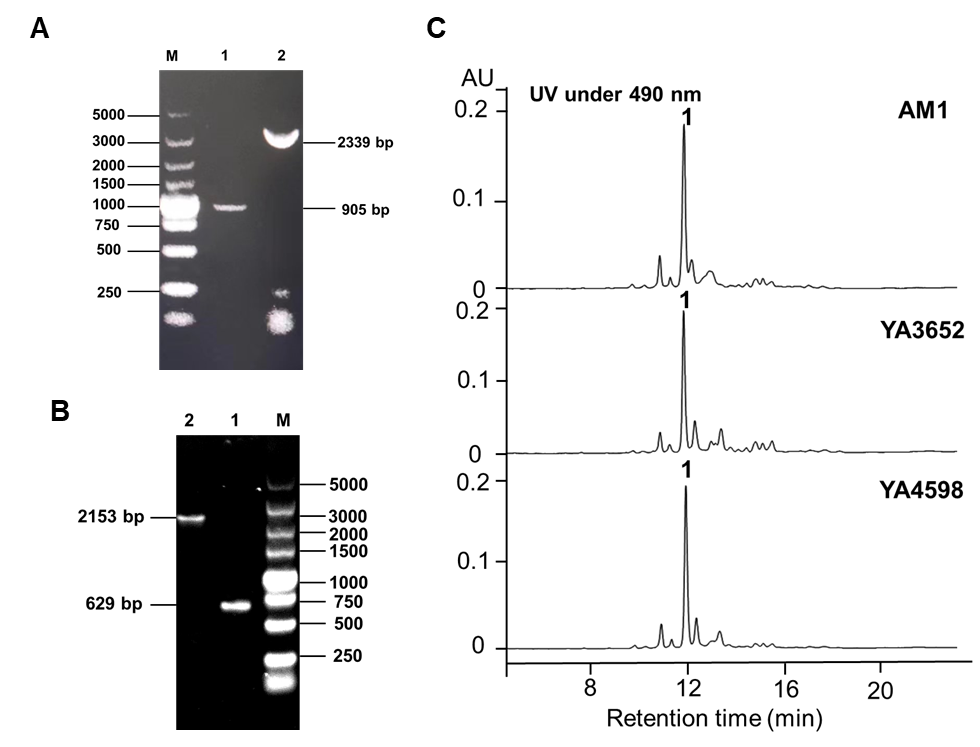


Figure S2. Analyses of the mutant strains YA4958 and YA3652. (A) Verification of genotype of YA4958 by using PCR, lane M: DNA ladder marker, lane 1: PCR fragments (2339 bp) from *M. extorquens* AM1 wild type, lane 2: PCR fragments (905 bp) from mutant strain YA4598. (B) Verification of genotype of YA3652 by using PCR, lane M: DNA ladder marker, lane 1: PCR fragments (2153 bp) from *M. extorquens* AM1 wild type, lane 2: PCR fragments (629 bp) from mutant strain YA3652. (C) HPLC analyses of metabolites from *M. extorquens* AM1 wild type, YA3652 and YA4598.

Table S1. The dry weight of *M. extorquens* derivative strains.

| Strain | Dry weight (g / L) |
| --- | --- |
| *M. extorquens* YAIO | 15.73 ± 0.52 |
| *M. extorquen* YAMZ1815 | 0.84 ± 0.07 |
| *M. extorquen* YAMZ1816 | 0.21 ± 0.04 |
